# *Vibrio vulnificus* pneumonia with multiorgan failure: a case report and review of the literature

**DOI:** 10.1186/s13256-023-03943-9

**Published:** 2023-05-19

**Authors:** Ishanya Abeyagunawardena, Dilshan Priyankara

**Affiliations:** Medical Intensive Care Unit, National Hospital of Sri Lanka, Colombo, Sri Lanka

**Keywords:** Pneumonia, *Vibrio vulnificus*, Case report, Primary sepsis, Sea water exposures

## Abstract

**Background:**

*Vibrio vulnificus* is a gram-negative bacterium causing three clinical syndromes namely, gastrointestinal symptoms, skin sepsis and primary sepsis. Primary sepsis exhibits mortality rates exceeding 50%, particularly in the immunocompromised. *Vibrio vulnificus* is transmitted via consumption of contaminated seafood and contaminated seawater skin exposure. We describe a rare case of an immunocompetent male presenting with an atypical *Vibrio vulnificus* infection, culminating in severe pneumonia requiring intensive care.

**Case presentation:**

A 46 year old Indian male dockyard worker, a non-smoker and teetotaler, of Indian origin presented to the emergency treatment unit of a tertiary care hospital in Sri Lanka, with fever, productive cough with yellow sputum, pleuritic chest pain and tachypnea for five days. He had no gastrointestinal or skin manifestations. His respiratory rate was 38 breaths/min, pulse rate was 120 bpm, blood pressure was 107/75 mmHg and pulse oximetry was 85% on air. Chest X-ray revealed consolidation of the left lung. Empiric intravenous Piperacillin-tazobactam and Clarithromycin were commenced after obtaining blood and sputum cultures. Over the next 24 h, his oxygen requirement rose and as he required vasopressor support, he was admitted to the intensive care unit. He was intubated and bronchoscopy was performed on day two, which demonstrated thick secretions from left upper bronchial segments. His antibiotics were changed to intravenous ceftriaxone and doxycycline following a positive blood culture report of *Vibrio vulnificus*. He was ventilated for ten days and his intensive care stay was complicated with a non-oliguric acute kidney injury, with serum creatinine rising up to 8.67 mg/dL (0.81–0.44 mg/dL). He developed mild thrombocytopenia with platelets dropping to 115 × 10^3^ /uL (150–450 × 10^3^/uL) which resolved spontaneously. Vasopressors were weaned off by day eight and the patient was extubated on day ten. He was discharged from intensive care on day twelve and made a full recovery.

**Conclusions:**

Pneumonia itself is an atypical manifestation of *Vibrio vulnificus* and furthermore, this patient was immunocompetent and did not exhibit the classical gastro-intestinal and skin manifestations. This case highlights the occurrence of atypical *Vibrio sp*. infections in patients with high exposure risks and the need for early supportive and appropriate antibiotic therapies.

## Background

*Vibrio vulnificus* is a gram-negative bacterium of the *Vibrio* genus which can cause severe and fatal disease [1]. It is transmitted via the consumption of contaminated fish and shellfish and through the skin when exposed to contaminated seawater. Host factors contribute significantly to the development of *V. vulnificus* infection and hence it is generally reported in patients with chronic liver disease or a history of alcohol abuse without documented liver disease and in patients with other chronic diseases such as diabetes mellitus, end-stage renal disease, rheumatoid arthritis and hemochromatosis [1]. Fatality rates are significantly higher in immunocompromised patients [2]. In this paper we aim to describe a rare case of a *V. vulnificus* infection culminating in severe pneumonia in an immunocompetent patient requiring intensive care.

## Case presentation

**Fig. 1:**
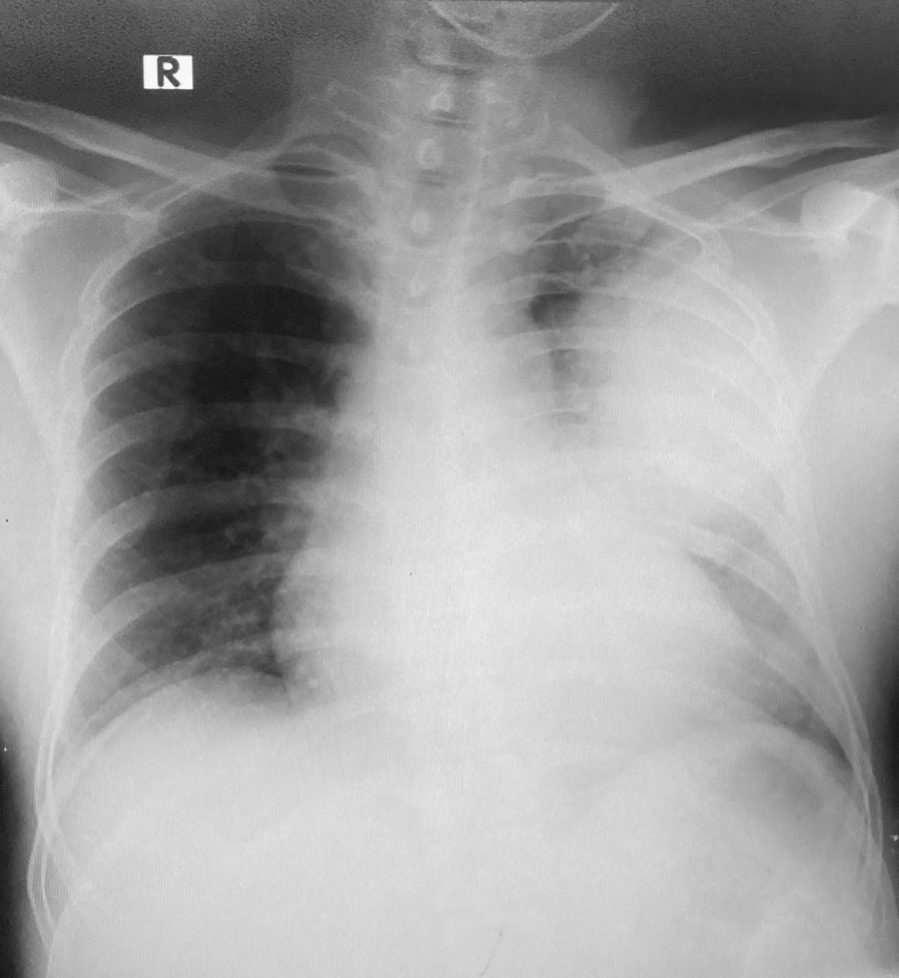
Chest X ray-AP on admission showing consolidations of the upper and middle zones of the left lung

A 46 year old male patient of Indian origin, presented to the emergency treatment unit (ETU) of a tertiary care hospital in Sri Lanka, with fever, productive cough with yellow sputum, pleuritic chest pain and tachypnea over five days. He had no gastrointestinal symptoms such as vomiting and diarrhea. He was previously fit and well with no prior medical illness of note. He was a non-smoker, teetotaler and worked at the dockyard as a welder. On admission, he was conscious, rational and febrile. He had a respiratory rate of 38 breaths per minute, a pulse oximetry (SpO2) of 85% on air and bilateral (predominantly left side) coarse crepitations on lung auscultation. His SpO2 improved to 92% with 3 l/min of Oxygen via nasal cannula. He had a pulse rate of 120 bpm and blood pressure of 107/75 mmHg. Abdominal examination was normal and there was no evidence of skin sepsis. Arterial blood gas revealed pH- 7.44, PaCO_2_- 23 mmHg, PaO_2_ 60 mmHg, HCO_3_^−^-16.5 mmol/l, lactate 1.7 mmol/l and a PaO_2_/ FiO_2_ ratio of 290 mmHg. The admission laboratory investigations revealed a white blood cell count of 3.79 × 10^3^/uL (4.00–10.00 × 10^3^/uL), a platelet count of 195 × 10^3^/uL (150–450 × 10^3^/uL), Haemoglobin of 13.7 g/dL (11–16 g/dL) and a serum creatinine of 1.8 mg/dL (0.81–0.44 mg/dL). An urgent chest X-ray (CXR) revealed consolidation of the upper and middle zones of the left lung (Fig. 1).

At the ETU, he was commenced on non-invasive ventilation (bilevel positive pressure ventilation IPAP-12cmH_2_O, EPAP-6 cmH_2_O, FiO_2_-40%) to support his breathing. Blood and sputum cultures were taken and subject to the availability of drugs, intravenous Piperacillin-tazobactam and Clarithromycin were commenced empirically. Subsequently, he was transferred to the high dependency area of the medical ward. Over the next 24 h he deteriorated clinically, requiring increasing oxygen requirements and vasopressor support and hence was admitted to the intensive care unit (ICU). On arrival to the ICU, he was intubated and initiated on lung protective ventilation.

His initial blood culture was positive for *Vibrio vulnificus* sensitive to Ceftriaxone, Cefuroxime, Amikacin and Ciprofloxacin and therefore, his antibiotics were changed to intravenous Ceftriaxone and Doxycycline. Sputum culture revealed no growth. His oxygen requirement gradually rose over the next few days to an FiO_2_ of 0.8%. Bronchoscopy was performed on day two of ICU care which demonstrated thick secretions from left upper bronchial segments. The secretions were cleared and samples were sent for gram stain and culture. COVID-19 polymerase chain reaction, melioidosis antibodies and retroviral screening were negative. The bronchoscopy sample did not reveal any positive gram stain or growth.

**Fig. 2:**
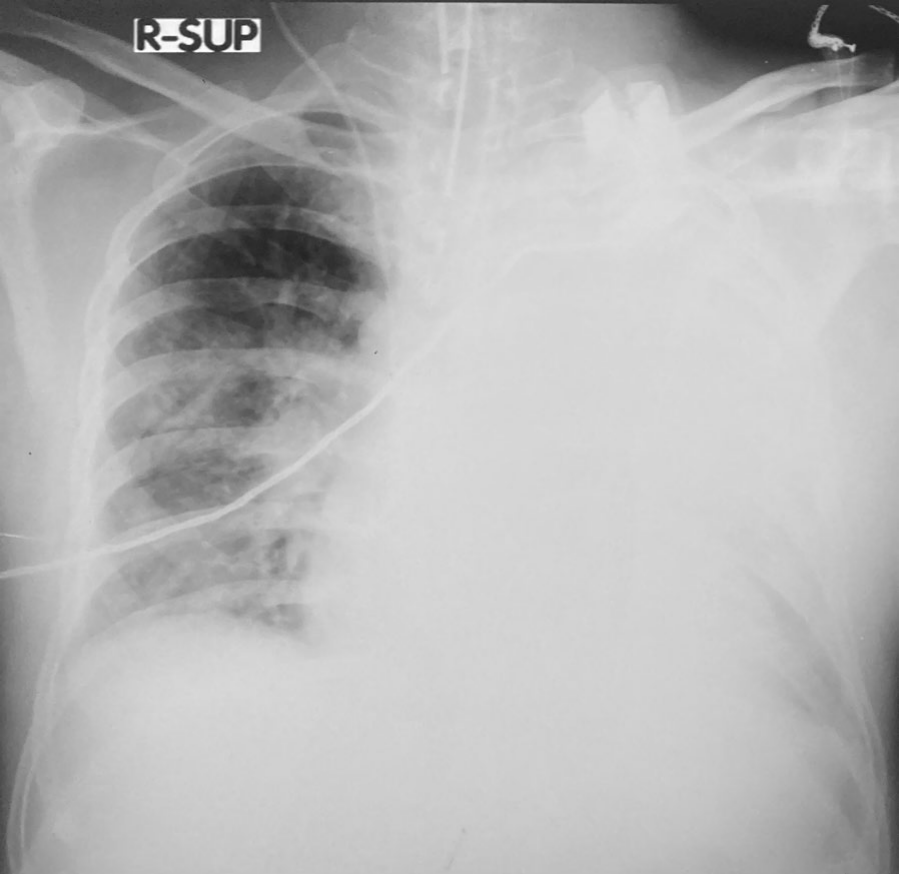
Repeat chest X ray on day 4 showing diffuse consolidation of the left lung

**Fig. 3:**
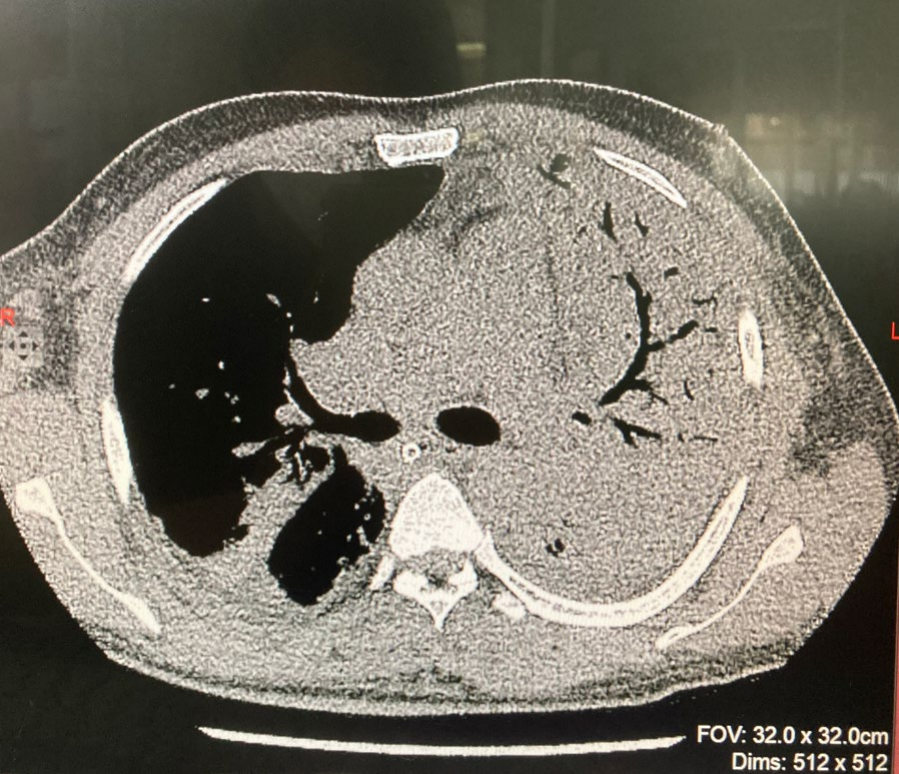
High resolution computed tomography chest showing dense consolidation of left lung

Repeated CXRs on day four and day seven showed persisting dense consolidations, mainly involving the left lung (Fig. 2). The high resolution computed tomography (HRCT) scan reported severe consolidation changes affecting the entire left upper lobe (apart from lingular segment), and the entire left lower lobe (apart from the lateral segment). Ground glass opacities and atelectatic changes were seen in the left lingular segment and lateral segment (Fig. 3).

The patient’s ICU stay was complicated with an non oliguric acute kidney injury with serum creatinine rising up to 8.67 mg/dL (0.81–1.44 mg/dL). He was also in septic shock requiring intravenous noradrenaline up to 1.2 mcg/kg/min which was weaned off by day eight. No other inotropes or vasopressors were required. In addition, he developed mild thrombocytopenia with platelets dropping to 115 × 10^3^ /uL (150–450 × 10^3^ /uL) with no bleeding manifestations. The drop in platelets was attributed to the sepsis and it spontaneously resolved, with no requirement of transfusions.

He was gradually weaned to bilevel positive airway pressure ventilation and his fever settled by day six of ICU stay. The patient was extubated on day ten to high flow nasal oxygen and discharged from intensive care on day twelve. Upon discharge to the medical ward, he was not on any oxygen or vasopressor support. He was discharged from the hospital two days later and regained full strength within two weeks, subsequently returning to his workplace.

## Discussion and conclusions

*V. vulnificus* is a potentially fatal pathogen with spread occurring faeco-orally and via the skin. Males have been observed to have more severe disease than females, the reason for which is yet unknown [1]. In addition, immunocompromised hosts are more likely to develop severe disease [2]. *V. vulnificus* infection manifests as three distinct syndromes: gastro-intestinal disease, wound infections and overwhelming primary sepsis. Sepsis is often accompanied by skin and soft tissue infections and has the highest mortality rates [1, 3]. It is a bacteremia with no definite route of infection, with the small intestine or the proximal colon being postulated as possible ports of entry. Infected individuals present with abrupt fever with chills and gastrointestinal symptoms with skin lesions, such as bullae, ecchymosis or rashes in the lower extremities [1]. Septic shock (systolic blood pressure < 90 mmHg), obtundation, lethargy, confusion and thrombocytopenia have also been reported in patients with primary septicaemia [3].

Atypical manifestations of *V. vulnificus* infection includes pneumonia, meningoencephalitis, peritonitis, pyogenic spondylitis, endometritis, septic arthritis, spontaneous bacterial peritonitis, endophthalmitis and keratitis [1, 3].

A case report of primary septicemia being complicated with diffuse pulmonary infiltrates has been reported in the literature [4]. In addition, a case report on a drowning victim with primary septicemia and pneumonia, with the isolation of lactose positive *Vibrio* in seawater has also been previously reported [5]. Another publication includes a letter to the editor of Annals of Internal Medicine detailing the story of a 60 year old male with a history of alcohol abuse, who fell into the waters of a harbor in Baltimore and subsequently contracted *Vibrio vulnificus* pulmonary infection. He had high fevers and infiltrates in the right lower lobe and left upper lobe. He also had a transient diarrhea lasting 72 h and was treated with Tetracycline and Gentamycin for 14 days [6].

Our case highlights the more atypical forms of presentation of *Vibrio vulnificus* infection. His occupation as a dockyard worker with daily exposures to seawater and seafood is likely to be the key factor which resulted in this infection. Notably, this patient was immunocompetent with no history of alcohol abuse or any chronic disease. He did not have any gastro-intestinal symptoms such as vomiting or diarrhea typical to this infection, throughout his disease course. Furthermore, no skin manifestations were noted and the infection manifested primarily as a severe pneumonia confined to the left lung. However, he exhibited other features of primary septicemia such as septic shock, thrombocytopenia and development of acute kidney injury.

Primary septicemia has been reported to be fatal in 60–75% of cases with the presence of hypotension within 12 h of admission being a poor prognostic factor (these patients have twice as high mortality rates) [3]. Our patient had a turbulent course of illness, being vasopressor and ventilator dependent within 2 days of admission. However, he responded well to treatment with Ceftriaxone and Doxycycline and was extubated after ten days, following which he made a full recovery. It has been reported in the literature that third-generation Cephalosporin with Doxycycline, or Quinolone with or without a third-generation Cephalosporin are potential treatment options for *V. vulnificus* infection [1]. It is also likely his premorbid good state of health contributed to his recovery.

In conclusion, *Vibrio vulnificus* sepsis is an infection with devastatingly high mortality rates requiring early supportive and appropriate antibiotic therapy to increase the likelihood of a positive outcome. Atypical *Vibrio sp.* infections should be kept in mind when treating patients with recent exposures to seawater and possible contaminated seafood, despite the lack of classical gastro-intestinal and skin symptoms. Patients with chronic disease and alcohol use are more prone to severe infections and should be identified as high risk, however, severe infections in the immunocompetent remains a very real possibility.

## Data Availability

Not applicable.

## Abbreviations

ETU: Emergency treatment unit
ICU: Intensive care unit
CXR: Chest X ray
HRCT: High resolution computed tomography
